# Cleansing efficacy of an oral irrigator with microburst technology in orthodontic patients—a randomized-controlled crossover study

**DOI:** 10.1007/s00784-023-05003-4

**Published:** 2023-04-06

**Authors:** Vera Wiesmüller, Manuel Kasslatter, Baran Zengin, Desiree Zotz, Vincent Offermanns, René Steiner, Adriano Crismani, Ines Kapferer-Seebacher

**Affiliations:** 1 Department of Conservative Dentistry and Periodontology, Medical University of Innsbruck, Anichstr. 35, 6020 Innsbruck, Austria; 2 Department of Orthodontic Dentistry, Medical University of Innsbruck, Anichstr. 35, 6020 Innsbruck, Austria; 3 Private oral surgery practice, 6122 Fritzens, Austria; 4 Department of Prosthetic Dentistry, Medical University of Innsbruck, Anichstr. 35, 6020 Innsbruck, Austria

**Keywords:** Oral irrigator, Biofilm(s), Orthodontic, Oral hygiene, Plaque index, Fixed orthodontic treatment

## Abstract

**Objectives:**

Orthodontic patients struggle with interdental cleaning calling for simpler mechanical devices to reduce the high plaque levels. The present study aimed to compare the cleansing efficacy of an oral irrigator with that of dental flossing in patients with fixed braces after 4 weeks of home-use.

**Materials and methods:**

The study design is a randomized and single-blinded cross-over study. After 28 days using the products at home, hygiene indices (Rustogi Modified Navy Plaque Index (RMNPI); gingival bleeding index (GBI)) were compared between test (oral irrigator) and control product (dental floss).

**Results:**

Seventeen adult individuals finalized the study. After 28 days of cleaning with the oral irrigator, RMNPI was 54.96% (46.91–66.05) compared to 52.98% (42.75–65.60) with dental floss (*p* = 0.029). Subgroup analysis revealed that the higher cleansing efficacy of the dental floss is attributable to buccal and marginal areas. GBI after the test phase with the oral irrigator was 12.96% (7.14–24.31) and statistically significantly higher compared to 8.33% (5.84–15.33) with dental floss (*p* = 0.030) which could be seen in all subgroups.

**Conclusions:**

Oral irrigators do not remove plaque and reduce gingival bleeding as efficiently as dental floss in easily accessible regions. However, in posterior regions, where the patients struggled with the application of dental floss, the oral irrigator showed similar results.

**Clinical relevance:**

Oral irrigators should only be recommended to orthodontic patients who cannot use interdental brushes and are not compliant with dental flossing.

## Introduction

Recent studies have shown that interdental cleaning in a high frequency (4 to 7 times per week) is associated with less carious lesions, less periodontal disease, and a lower number of missing teeth [1]. Interdental cleaning devices should be user-friendly, effective in removing dental biofilm, should access the teeth, respectively root surfaces, on all interproximal facets and must not harm soft or hard tissues. Although dental floss is the most recommended interdental cleaning device, research has shown that interdental brushes are to be favored concerning efficacy [2]. The compliance using dental floss is low, and many patients fail to implement flossing into their daily routine due to the challenging correct usage [3]. Therefore, flossing cannot be recommended other than for sites of gingival and periodontal health, as often seen in younger patients where interdental brushes will not pass through the interproximal area without trauma [2, 4]. Otherwise, interdental brushes are the device of choice for interproximal plaque removal [2, 4].

In orthodontic patients, fixed braces promote supra- and subgingival accumulation of biofilm by impeding oral hygiene resulting in an altered oral microbiome, enamel decalcification, and gingivitis [5–7]. It was recently shown that patients with upper and lower multibracket appliances are affected significantly more frequently by gingivitis (65%) and white spot lesions (30%) [8].

In the predominantly young orthodontic patients, interdental spaces are often too narrow to use interdental brushes and flossing is challenging and time-consuming. There is a wide range of interdental cleaning devices, which should facilitate the process of interdental cleaning, since impracticability is seen as an important cause for incompliance. Oral irrigators are easy to use even in the presence of orthodontic braces and are therefore favored by many patients [9]. Most oral irrigators use a stream of water only to mechanically remove plaque from interproximal areas but there are also oral irrigators which use a mixture of air and water, called microburst technology. Although in vitro trials have provided promising results, there is only a very small number of independent randomized controlled trials which tested the effectiveness of oral irrigators with microburst technology [10, 11]. Limited evidence in patients with periodontal health and/or gingivitis has shown that microburst technology in oral irrigators may improve gingival health and reduce gingival bleeding indices comparable to dental floss, while reduction of visible plaque was not demonstrable to the same extent [12–14]. Clinical data in periodontal patients in supportive periodontal therapy showed a reduction in bleeding on probing over a long-term period of 6 months [9, 15]. Current evidence concerning irrigators in orthodontic patients is also very limited and reports inhomogeneous results regarding efficacy [16]. A study designed for testing an oral irrigator with microburst technology in orthodontic patients has not yet been conducted.

Since experience shows that effective oral hygiene with fixed orthodontic appliances is time-consuming and tedious, we aim to find simpler mechanical devices for interdental cleaning. The objective of the present randomized and single-blinded cross-over study was to compare the cleansing efficacy of microburst technology with that of dental flossing in orthodontic patients with fixed braces after 4 weeks of home-use. The null hypothesis states no difference between the two methods.

## Material and methods

This study was approved by the Ethics committee of the Medical University of Innsbruck, Austria (ID AN 5123). The study was conducted in accordance with the 1964 Helsinki declaration and its later amendments. Prior to inclusion all subjects signed an informed written consent.

### Study subjects

Twenty adult subjects of the University Hospital of Orthodontic Dentistry, Innsbruck, Austria, were recruited in the period from November 5th, 2020, to January 9th, 2021. Inclusion criteria were fixed braces attached buccally at a minimum of four teeth per quadrant and existing contact points between all teeth. Exclusion criteria were pregnancy, minority, oral or systemic diseases other than gingivitis, and the need for frequent drug consumption to prevent hormonal or drug-induced distortion, especially of the gingival index. Teeth with ceramic restorations and implants were excluded from analysis due to different plaque adhesion compared to natural teeth. Data collection was performed from January 26th, 2021, to June 30th, 2021.

### Clinical intervention

The cleansing efficacy of the microburst technology (*Airfloss*®, Philips, Hamburg, Germany) versus interdental cleaning with dental floss (*Superfloss*®, Oral-B, Boston, USA) was evaluated in a randomized-controlled, examiner-blinded, crossover study.

The study design consisted of four appointments for each subject. At the first appointment all probands were thoroughly informed about the study protocol, inclusion and exclusion criteria were surveyed, and an informed consent was signed. Moreover, baseline hygiene indices were evaluated using the Rustogi Modified Navy Plaque Index (RMNPI) [8] after plaque disclosing (*2Tone*, Young, Earth City, MO, USA) and the gingival bleeding index after Ainamo and Bay (GBI) [17].

The Rustogi Modified Navy Plaque Index (RMNPI) splits every buccal and lingual tooth surface into nine sections (A–I) that are assessed for the presence or absence of plaque. The index allows to draw a distinction between marginal areas of the teeth (A–C), interdental areas (D, F), or overall surface areas (A–I). RMNPI is calculated as the percentage of biofilm adhering sites to measured sites. For the assessment of the gingival bleeding index (GBI), a periodontal probe (PCP 12, Hu Friedy, Chicago, USA) was inserted into the gingival sulcus to decide dichotomously at six sites per tooth (mesiobuccal–buccal–distobuccal–mesiolingual–lingual–distolingual) if bleeding occurred or not. The percentage of bleeding sites to measured sites was calculated. Teeth that were not integrated in the fixed orthodontic treatment were excluded. All examinations were conducted by one trained examinator.

Randomization of the test products was computer-generated prior to investigation using *Microsoft*® Office Excel and was conducted by study assistants, who also thoroughly instructed the subjects to use the products through hands-on training to ensure that the examiner did not know which product was used and so could collect the data blindly. According to the manufacturer’s protocol (*Airfloss*®, Philips, Hamburg, Germany), the oral irrigator with microburst technology was filled with water and activated once per interdental space with the default setting of three sprays per activation. The control product (*Superfloss*®, Oral-B, Boston, USA) was instructed to thread from buccal below the orthodontic wire and place it around the tooth in a C-shaped manner to clean in apico-coronal direction. Regarding toothbrushing, the participants were asked to stick to their usual routine and product. After detailed instruction with the first randomized assigned test product, professional tooth cleaning was conducted with an air-polishing device (*Airflow*® prophylaxis master and *Airflow*® Plus powder; both EMS, Nyon, CH) and if needed sonic scalers and rubber cups with polishing paste (*Cleanic*®, Kerr, Bioggo, CH).

After 28 days using the first test product, the study subjects presented for their second visit. The hygiene indices and inclusion/exclusion criteria were surveyed again. After a wash out phase of 28 days where the probands were practicing their usual oral hygiene procedures, they presented for the third visit. Again, plaque was disclosed, and the subjects were thoroughly instructed to use the second product followed by a professional dental cleaning. In analogy to the first test phase, the subjects used the product for 28 days and then presented for examination of the plaque and gingival index in the context of the fourth and final appointment of the study.

### Statistical analysis

The sample size calculation was based on mean values and standard deviations of overall plaque scores provided by Heiß-Kisielewsky et al. comparing the cleansing efficacy of microburst technology (*Airfloss*®, Philips, Hamburg, Germany) to dental flossing [14]. The mean plaque score applying the oral irrigator was 153.91 plaque-positive sites and for dental flossing 145.95 plaque-positive sites with a mean standard deviation of 10.55. Sample size calculation for dependent samples, a power of 80%, *α* = 0.05 resulted in a sample size of 16, and with a presumed drop-out rate of 25% a sample size of *n* = 20.

On a proband-level, RMNPI values were calculated as the total number of areas with plaque present divided by the total number of sites scored and were then compared between the two tooth brushing procedures by Wilcoxon signed-rank test. The gingival bleeding index was calculated in the same manner. If not stated otherwise, median and interquartile range are given. Significance level was set at *p* < 0.05.

## Results

Twenty individuals were recruited. Seventeen participants (seven females and ten males) finished the study with a mean age of 27.12 ± 9.23 (range 18–49) years. The drop-out rate was 15%. One participant quit because of scheduling difficulties; two participants were excluded because of antibiotic treatment during the test phase. A total of 446 teeth were included in this study.

### Plaque scores

**Fig. 1:**
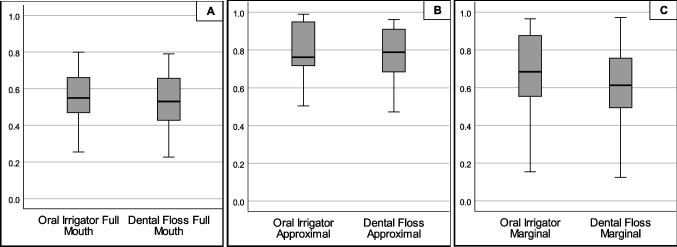
Rustogi Modified Navy Plaque Index (RMNPI) after 28 days of home-use of an oral irrigator in comparison to dental floss. Rustogi modified plaque index splits every buccal and lingual tooth surface into nine sections (A–I) and was calculated as percentage of biofilm adhering sites to measured sites of **A** all tooth surfaces (A–I), **B** approximal surfaces (D, F), and **C** marginal surfaces (A–C) of the teeth

At baseline, the median of overall RMNPI was 61.35% (53.29–69.56). After 28 days of interdental cleaning with microburst technology, the median of overall RMNPI was 54.96% (46.91–66.05) and statistically significantly higher than after 28 days of interdental cleaning with the control procedure dental flossing (median of overall RMNPI 52.98%; range 42.75–65.60) (*p* = 0.029) (see Fig. 1). Compared to baseline, a statistically significant difference could be seen after using the dental floss (*p* = 0.020), but not after using the oral irrigator (*p* = 0.105).

Subgroup analysis revealed that the higher cleansing efficacy of the dental floss is mainly attributable to buccal and marginal areas (see Fig. 1) and not to approximal areas. There was a statistically significantly lower plaque index after 28 days of dental flossing compared to microburst technology on marginal areas (median 61.25% and 68.45%, respectively; *p* = 0.010) but not on approximal areas (median 78.85% and 76.19%, respectively; *p* = 0.215). Additionally, the difference was more pronounced on buccal than on lingual/palatal surfaces (see Table 1).

**Table 1:** Plaque and bleeding levels after 1 month of home-use. The Rustogi modified plaque index splits every buccal and lingual tooth surface into nine sections (A–I) and was calculated as percentage of biofilm adhering sites to measured sites. Gingival bleeding was calculated dichotomously at 6 sites per tooth as percentage of bleeding sites to measured sites. Data was presented using median and interquartile ranges

	Microburst technology	Dental flossing	*p* value
Full mouth			
RMNPI (%)	54.96% (46.91–66.05)*	52.98% (42.75–65.60)*	0.029
Gingival bleeding index (%)	12.96% (7.14–24.31)*	8.33% (5.84–15.33)*	0.030
Approximal sites			
RMNPI (%)	76.19% (71.68–94.85)	78.95% (68.48–91.0)	0.215
Gingival bleeding index (%)	16.35% (8.04–23.96)*	9.38% (6.73–15.38)*	0.019
Approximal buccal sites			
RMNPI (%)	78.95% (71.93–98.04)	78.57% (75.00–89.58)	0.308
Gingival bleeding index (%)	19.23% (7.14–21.43)	9.26% (6.52–12.50)	0.064
Approximal lingual/palatal sites			
RMNPI (%)	78.85% (71.43–92.86)	78.57% (64.58–92.31)	0.865
Gingival bleeding index (%)	13.46% (8.93–27.08)	9.09% (7.14–16.00)	0.074
Marginal sites			
RMNPI (%)	68.45% (55.36–87.65)*	61.27% (49.40–75.68)*	0.010
Gingival bleeding index (%)	12.96% (7.14–24.31)*	8.33% (5.84–15.33)*	0.030
Marginal buccal sites			
RMNPI (%)	58.97% (47.62–76.92)*	51.90% (41.77–60.00)*	0.025
Gingival bleeding index (%)	12.82% (5.95–17.95)	7.69% (5.13–11.11)	0.057
Marginal lingual/palatal sites			
RMNPI (%)	75.00% (63.10–93.83)	75.00% (60.71–83.95)	0.250
Gingival bleeding index (%)	14.10% (8.33–31.94)	8.54% (7.14–14.67)	0.051
Anterior teeth			
RMNPI (%)	59.44% (46.30–64.35)*	53.70% (42.59–60.19)*	0.019
Gingival bleeding index (%)	9.72% (5.56–20.83)*	5.56% (2.78–6.94)*	0.012
Posterior teeth			
RMNPI (%)	55.16% (47.57–70.09)	50.69% (46.53–67.46)	0.263
Gingival bleeding index (%)	16.67% (7.53–28.89)	12.50% (7.53–28.89)	0.056

*RMNPI*, Rustogi Modified Navy Plaque Index; %, percent; *, *p* value < 0.05

### Gingival bleeding index

At baseline, the median of GBI was 26.45% (range 14.49–31.55). After 28 days of interdental cleaning with the oral irrigator, GBI was 12.96% (7.14–24.31) and statistically significantly higher compared to 8.33% (5.84–15.33) after interdental cleaning with dental floss (*p* = 0.030). Both tested products, the dental floss and the oral irrigator, reduced gingivitis in the statistically significantly compared to baseline (*p* < 0.005). The same applies to all subgroup analyses.

**Fig. 2:**
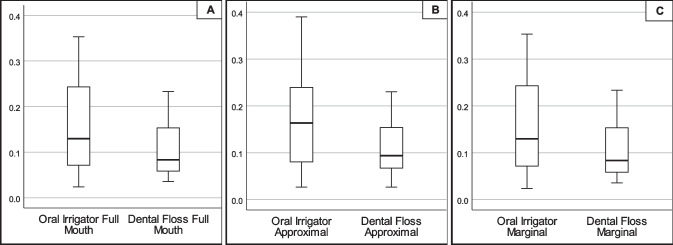
Gingival bleeding index after 28 days of home-use of an oral irrigator in comparison to dental floss. Gingival bleeding index was calculated dichotomously as percentage of bleeding sites to measured sites of **A** all tooth surfaces, **B** approximal surfaces, and **C** marginal surfaces of the teeth

Subgroup analysis revealed that unlike the plaque index, gingival bleeding was statistically significantly different not only at marginal sites but also at approximal sites (see Fig. 2). There was a statistically significantly higher gingival bleeding index after 28 days of home-use of the oral irrigator compared to dental flossing on marginal areas (12.96% and 8.33%, respectively; *p* = 0.030) and on approximal areas (16.35% and 9.38%, respectively; *p* = 0.019). Again, the difference was more pronounced on buccal than on lingual/palatal surfaces. The gingival bleeding index was also statistically significantly higher in anterior teeth after using the oral irrigator compared to dental flossing (median 9.72%, range 5.56–20.83 and median 5.56%, range 2.78–6.94, respectively; *p* = 0.012) but not in posterior teeth (median 14.10%, range 8.33–31.94 and median 8.54%, range 7.14–14.67; respectively; *p* = 0.056).

## Discussion

Home oral care is a daily challenge for patients with brackets for many months or even years. Interdental cleaning is particularly difficult, since the interdental spaces in young adults, most of whom have periodontal health, are not accessible to interdental brushes. The handling of dental floss is complicated by the orthodontic wires. Therefore, the most common side effects of fixed orthodontic treatment include gingivitis and initial caries [6–8]. In order to be able to make oral hygiene recommendations for patients with fixed orthodontics that are evidence-based and as simple as possible in the future, we are testing the effectiveness of various interdental cleaning devices in orthodontic patients in a series of clinical studies. The aim of the present study was to compare the cleansing efficacy of oral irrigators in orthodontic patients, which seem to be an easy-to-use alternative to dental flossing.

In the present study, we decided to use the Rustogi Modified Navy Plaque Index [18], a dichotomous index evaluating plaque presence or absence in nine areas on buccal and lingual surfaces which is a quite time-consuming procedure. It allows to assess plaque levels on a full-mouth level, but also subgroup analyses for smooth surfaces, interdental and gingival margin areas separately. A disadvantage of dichotomous plaque indices is the variability of plaque amounts which requires intense examiner calibration in the case of multiple investigators or—as in our study—one trained investigator measuring all plaque indices. The authors present two main arguments for the use of dichotomous plaque indices. Firstly, statistical analyses of ordinal indices are difficult to translate to daily routine. Secondly, the most frequent way to analyze ordinal plaque scores is to treat them as metric variables, calculating mean ± standard deviation or median and interquartile range of all measured sites and using non-/parametric statistical tests, which is questionable from a statistical point of view [19–21].

Baseline plaque indices in the cohort at hand were high (median RMNPI 61.35%; range 53.29–69.56), although not higher than in previous studies of cohorts without fixed orthodontics [22, 23]. Assessment of approximal sites only prior to intervention showed an even higher score of 83.93% (77.68–95.0) confirming the necessity for improving interdental cleaning in orthodontic patients.

Both tested products, the dental floss and the oral irrigator, reduced gingivitis in the interdental regions statistically significantly compared to baseline (*p* < 0.005). However, dental flossing resulted in less gingival bleeding than cleaning with the oral irrigator, not only at approximal sites but also at marginal sites (*p* < 0.05) (see Fig. 2).

Previous clinical trials investigating oral irrigators showed promising results, for instance Bertl et al. noted a reduction of approximal bleeding compared to baseline from 32.6 to 23.1% (*p* = 0.027) after 12 weeks of usage [9]. The very limited data available assessing oral irrigations in orthodontic patients is inhomogeneous. In one study, the superiority of an oral irrigator with an orthodontic tip over dental floss in reducing gingival index was demonstrated (*p* < 0.001) [24], while in another examination a conventional oral irrigator could not show any additional benefit to brushing teeth after 6 months (*p* = 0.568) [16]. This should be a call to the industry for products specifically adapted to fixed appliances.

The presented results likewise show that dental floss reduced gingival bleeding statistically significantly more than the oral irrigation in overall surfaces (*p* = 0.030) as well as approximal (*p* = 0.019) and marginal surfaces (*p* = 0.030). However, no differences were shown between the two products in the posterior region, where the application of the dental floss is particularly challenging (*p* = 0.056).

The superiority of dental flossing was not that pronounced for the plaque index. The results showed a statistically significantly lower RMNPI compared to oral irrigation only at marginal (*p* = 0.010) but not at approximal sites (*p* = 0.215) (see Fig. 1). The present findings confirm a review of the literature which describes no additional benefit of using an oral irrigator compared to dental floss in terms of reduction of visible plaque [12, 16].

Application of dental floss seems to be easier on the buccal side than lingually/palatally, as the plaque scores were statistically significantly lower on buccal than on lingual sites (median RMNPI 51.90%, range 41.77–60.00 and 75.00%, range 60.71–83.95, respectively). This is consistent with our personal observations. Patients find it relatively easy to apply and move the floss in a C-shape at both adjunctive teeth at buccal sites, but they tend to keep the floss straight and still lingually/palatally. The oral irrigator is used from the buccal side only. Additional usage from lingual could reduce plaque scores in the lingual interdental spaces. However, for this purpose, the oral irrigator would have to be turned upside down which results in loss of function. A particularly noticeable difference was seen by comparing front to posterior teeth. The dental floss removed statistically significantly more plaque in the front region (*p* = 0.019), while a comparison of the dental floss and the oral irrigator in premolar and molar regions showed no statistically significant difference (*p* = 0.263). The authors again explain the lack of difference between dental floss and oral irrigator in the posterior region with the difficult access and overview regarding the posterior teeth.

Limitations of the study design could be a possible reduction of the gingival bleeding index because of the professional cleaning reducing the comparability to the baseline GBI. In order to achieve alignment of the first and second test phase, a wash-out phase of 28 days was selected, in which oral hygiene habits as before the intervention were maintained. Furthermore, the number of participants of the present study is rather low and inhomogeneous regarding gender and age. The seven females and ten males showed no significant difference in baseline hygiene indices, nor in plaque and gingival indices after the test phases when compared in groups regarding gender (*p* > 0.05). The crossover study design, where each participant tests both test products after a wash-out phase, compensated certain inhomogeneities in the study cohort since the total study cohort was used for comparison.

Since the guidelines of the local ethics committee do not approve the inclusion of adolescent patients in studies that can be carried out on adults, it is necessary to investigate whether these results can also be confirmed for adolescent patients, who account for a large proportion of orthodontic patients.

In conclusion, to date, oral irrigators are still in need of substantial technical improvement and do not remove plaque and reduce gingival bleeding as efficiently as dental floss in regions that were easy to reach. However, in posterior regions, where the patients struggled with the application of dental floss, the oral irrigator showed similar results. The approach to facilitate effective tooth cleaning in orthodontic patients should be pursued to reduce the very high plaque levels.
